# Inhibition of poly(ADP-ribose) Polymerase Interferes with *Trypanosoma cruzi* Infection and Proliferation of the Parasite

**DOI:** 10.1371/journal.pone.0046063

**Published:** 2012-09-25

**Authors:** Salomé C. Vilchez Larrea, Teemu Haikarainen, Mohit Narwal, Mariana Schlesinger, Harikanth Venkannagari, Mirtha M. Flawiá, Silvia H. Fernández Villamil, Lari Lehtiö

**Affiliations:** 1 National Institute for Genetic Engineering and Molecular Biology (INGEBI-CONICET), University of Buenos Aires, Buenos Aires, Argentina; 2 Biocenter Oulu and Department of Biochemistry, University of Oulu, Oulu, Finland; 3 Pharmaceutical Sciences, Department of Biosciences, Abo Akademi University, Turku, Finland; University of South Alabama, United States of America

## Abstract

Poly(ADP-ribosylation) is a post-translational covalent modification of proteins catalyzed by a family of enzymes termed poly(ADP-ribose) polymerases (PARPs). In the human genome, 17 different genes have been identified that encode members of the PARP superfamily. Poly (ADP-ribose) metabolism plays a role in a wide range of biological processes. In *Trypanosoma cruzi*, PARP enzyme appears to play a role in DNA repair mechanisms and may also be involved in controlling the different phases of cell growth. Here we describe the identification of potent inhibitors for *T. cruzi* PARP with a fluorescence-based activity assay. The inhibitors were also tested on *T. cruzi* epimastigotes, showing that they reduced ADP-ribose polymer formation *in vivo*. Notably, the identified inhibitors are able to reduce the growth rate of *T. cruzi* epimastigotes. The best inhibitor, Olaparib, is effective at nanomolar concentrations, making it an efficient chemical tool for chacterization of ADP-ribose metabolism in *T. cruzi*. PARP inhibition also decreases drastically the amount of amastigotes but interestingly has no effect on the amount of trypomastigotes in the cell culture. Knocking down human PARP-1 decreases both the amount of amastigotes and trypomastigotes in cell culture, indicating that the effect would be mainly due to inhibition of human PARP-1. The result suggests that the inhibition of PARP could be a potential way to interfere with *T. cruzi* infection.

## Introduction

The protozoan pathogen *Trypanosoma cruzi* (family *Trypanosomatidae*, order *Kinetoplastida*) is the causative agent of American Trypanosomosis or Chagas' disease, with over 15 million people infected only in Latin America and 12,500 deaths reported each year [1]. No vaccines have been developed to date to prevent American Trypanosomosis and only a few treatments are available. Beznidazole and Nifurtimox are the most commonly used drugs to treat Chagas' disease, but these are effective only during the acute phase of the illness, stage in which only a small percentage of the cases are detected due to unspecific symptoms. The few drugs available have proven to be inadequate, since effective therapeutic doses display high toxicity, causing harsh side effects. This, unfortunately, leads many patients to abandon the treatment [2].

In an effort to characterize the metabolism of *T. cruzi*, we found out that the protozoan parasite has an enzyme displaying similarities to human poly(ADP-ribose) polymerases (PARPs) recently renamed as diptheria toxin like ADP-ribose transferases (ARTDs) (EC 2.4.2.30) [3]. PARP catalyzes the transfer of an ADP-ribose moiety from NAD^+^ to the target protein or to the elongating polymer chain. Typically, PARP enzymes also catalyze an automodification reaction. Several proteins contain the characteristic PARP domain in higher eukaryotes and currently 17 members are included in the human PARP superfamily. These PARP enzymes are involved in diverse cellular processes such as signaling mechanisms in DNA damage, chromatin modification, transcription, cell death pathways, and mitosis [4]. The critical role played by human PARP-1 (hPARP-1) in DNA damage signaling and repair is its most extensively studied function. hPARP-1 has received a lot of interest as a therapeutic target in the pharmaceutical industry after the discovery that the growth of transformed cancer cells deficient in BRCA1/2 can be inhibited by hPARP-1 inhibitors alone [5]. This has accelerated the development of inhibitors and compounds from several companies have entered clinical trials in the past few years [6].

In contrast to what has been reported for higher eukaryotes, there is only one PARP present in *T. cruzi* (TcPARP). The initial characterization of the metabolism of poly ADP-ribose (pADPr) in *T. cruzi* showed that TcPARP is, like hPARP-1, activated by DNA strand breaks *in vitro* and *in vivo* when parasites are exposed to DNA damaging agents such as H_2_O_2_ and UVC radiation [7], [8]. The mechanism of activation is likely different from that of hPARP-1, as the trypanosomatid enzyme does not contain the zinc finger domains responsible for the DNA nick detection. Our earlier results indicated that the inhibition of PARP would impact negatively on the growth of *T. cruzi* epimastigotes, although in that case a low potency general PARP inhibitor, 3-aminobenzamide (3-AB), was used at a high 10 mM concentration [8]. These findings prompted us to study the inhibition of the enzyme in more detail as a plausible approach in the improvement of current therapeutics available for the treatment of the infection caused by *T. cruzi*. Here we report the characterization and inhibition of TcPARP *in vitro*. The most potent enzyme inhibitors discovered were also tested in *T. cruzi* parasite cultures to assess how they affect pADPr formation after a genotoxic stimulus and how they affect the growth of the parasite. Our results demonstrated that the inhibitors reduce pADPr formation with varying efficacies when the parasites are subjected to DNA damaging agents. Importantly, the best inhibitors were able to diminish the growth rate of *T. cruzi* epimastigotes even at nanomolar concentrations. The most potent inhibitor, Olaparib, also drastically reduced the number of intracellular amastigotes in human cell culture.

## Methods

### Protein expression

TcPARP was expressed as a 6xHis-tag protein using the pET-22b+ expression vector [7]. The vector was transformed into Rosetta 2(DE3) strain and the expression was carried out in Terrific Broth (TB) autoinduction media supplemented with 8 g/l glycerol, 100 µg/mL ampicillin, and 34 µg/mL chloramphenicol. The culture was grown at 37°C until OD_600_ reached 0.5 and induction was performed at 27°C overnight. The cells were harvested and suspended in Lysis buffer (50 mM HEPES pH 7.4, 0.5 M NaCl, 10% glycerol, 10 mM imidazole, 0.5 mM TCEP) and stored at −20°C.

### Protein Purification

Cells were thawed and supplemeted with 0.5 mg/ml of lysozyme (Sigma-Aldrich), 5 U/ml of benzonase (Sigma-Aldrich), and EDTA-free Complete protease-inhibitor cocktail (Roche) according to the manufacturer's instructions. The cells were incubated on ice for 30 minutes and the cell lysis was completed by sonication. The cell debris was removed by centrifugation (30000×*g*, 30 min) and the soluble fraction was filtered through a 0.45 µm syringe filter. The cleared lysate was loaded onto a Ni-charged 1 ml HisTrap FF crude column (GE Healthcare) equilibrated with Lysis buffer and washed with 30 column volumes of Lysis buffer and 10 column volumes of buffer containing 50 mM HEPES pH 7.4, 0.5 M NaCl, 10% glycerol, 25 mM imidazole, 0.5 mM TCEP. The protein was eluted with 50 mM HEPES pH 7.4, 0.5 M NaCl, 10% glycerol, 250 mM imidazole, 0.5 mM TCEP. Further purification was carried out with HiLoad 16/600 Superdex 200 prep grade column (GE Healthcare) using 50 mM HEPES pH 7.4, 0.5 M NaCl, 10% glycerol, and 0.5 mM TCEP. The final protein preparation was frozen in liquid nitrogen immediately after purification and stored at −70°C.

### Optimization of the homogenous activity assay

Enzymatic activity was measured using a fluorometric assay, which quantifies the remaining NAD^+^ after the enzymatic reaction [9], [10]. Briefly, TcPARP was incubated with 500 nM NAD^+^ in a 96-well U-bottom black propylene plate (Greiner BioOne) in a final volume of 50 µl. The reaction was carried out at 25°C with shaking at 300 rpm using PST-60 HL plus Thermo Shaker (Biosan, Riga, Latvia). The reaction was terminated by the addition of 20 µl of 2 M KOH and 20 µl of 20% acetophenone in ethanol, and plate was incubated at room temperature for 10 minutes. Next, 90 µl of 88% formic acid was added and the plate was incubated at room temperature for 20 minutes. The fluorescence was measured on a Fluoroskan Ascent FL (Thermo Labsystems, Waltham, MA) using excitation at 355 nm and emission at 460 nm. The activity assay was optimized for TcPARP by assaying the effect of pH, DNA concentration, and various additives for the activity of the protein.

### Screening of inhibitors

A small in-house library of known PARP inhibitors was screened to evaluate their inhibitory effect against TcPARP. The inhibitor stock solutions were prepared at 10 mM in 100% DMSO and stored at −20°C, except in the case of 3-aminobenzamide, which was prepared at a stock concentration of 1 M in 100% DMSO. The compounds were screened in duplicates at 10 µM and 1 µM concentrations with separate controls for each including only NAD^+^ and the compound, but not TcPARP. The protein concentration in the reactions was 10 nM.

### Western blot

The inhibitory effect of the best inhibitors was confirmed by Western blot. TcPARP (150 nM) was incubated in a mixture of 4 µM NAD^+^, 1 µM of biotinylated NAD^+^ (bioNAD^+^) (Trevigen) and 1 µM of inhibitor in the optimized assay buffer at room temperature for 1 hour. Positive control was done in the absence of any inhibitor, and the negative control in the absence of TcPARP. The reaction was stopped by adding Laemmli buffer and heating the samples for 5 min at 75°C. Lysozyme was added as a loading control. The samples were separated with SDS-PAGE and blotted onto a nitrocellulose membrane (Whatman). Blocking was performed at 4°C overnight followed by 1 hour at room temperature with 1% casein (BioRad). The proteins modified by biotinylated pADPr were detected using 1∶15000 streptavidin conjugated horseradish peroxidase (PerkinElmer).

### Measurement of inhibitor potencies

IC_50_ values were determined for the best inhibitors based on the inhibitor screening. The IC_50_ value for the first generation PARP inhibitor 3-AB was determined as a reference value. The separate data points were measured in quadruplicates. The assay setup was similar to the inhibitor screening, but the incubation time was set so that the substrate conversion in the control wells was approximately 20%. The fluorescence signal was converted to inhibition percentage and fitted using sigmoidal dose-response fitting with variable slope (Graphpad Prism). The curves were extrapolated to 0% and 100% of inhibition by setting their inhibitor concentration 2 log units below or above the first and the last inhibitor concentration, respectively.

### Homology modeling and structural analysis

Sequences in the protein data bank were searched for homologues of TcPARP (Uniprot id: Q4PQV7). Homologous high quality structures of hPARP-1 (PDB code 3GJW [11]), human PARP-2 (hPARP-2) (PDB code 3KJD [12]) and human PARP-3 (hPARP-3) (PDB code 3C4H [13]) were selected and aligned with TcPARP using ClustalW [14] with minor manual editing based on the superposed structures. Alignment was analyzed using Aline [15]. TcPARP was modeled using the hPARP-1 structure and multiple sequence alignment using Modeller [16]. Superpositions used in the text and figures were made using the SSM superposition algorithm in COOT [17], [18]. Structural representations were made using Pymol [19].

### Parasite cultures and *in vivo* inhibition of *Trypanosoma cruzi* PARP

*T. cruzi* epimastigote forms (CL Brener strain) were cultured at 28°C in liver infusion tryptose (LIT) medium (5 g/L liver infusion, 5 g/L, bacto-tryptose, 68 mM NaCl, 5.3 mM KCl, 22 mM Na_2_HPO_4_, 0.2% (w/v) glucose and 0.002% (w/v) hemin) supplemented with 10% (v/v) FCS, 100 U/ml penicillin and 100 mg/l streptomycin. Cell viability was assessed by direct microscopic examination.

*Trypanosoma cruzi* epimastigotes were grown in LIT complete medium for 4 days up to a parasite density of 10^7^ parasites/ml. Parasites were collected by centrifugation at 750×*g* for 5 minutes and resuspended in PBS-Glucose 2%. *T. cruzi* epimastigotes were pre-incubated in the presence of inhibitors for 30 minutes and treated with 300 µM hydrogen peroxide for 10 minutes. Cells were harvested by centrifugation at 1500×*g*, washed with PBS and resuspended in lysis buffer (50 mM Tris–HCl pH 8.0, 1.0 mM EDTA, 10% (v/v) glycerol, 10 mM 2-mercaptoethanol). Protease inhibitors 1 µg/mL trans-epoxysuccinyl-L-leucylamido(4-guanidino) butane (E-64), 1 mM pepstatine A, 1 mM phenylmethylsulfonylfluoride (PMSF), and 0.1 mM Na-tosyl-L-lysine chloro-methyl ketone (TLCK) and inhibitor 3-AB (1 mM) were added. Cells were lysed with an Ultrasonic Processor Model W385 Sonicator (Heat Systems-Ultrasonic Inc. Plainview) and the whole extract obtained was used for Western blot. 30 µg of protein were run on 10% SDS–PAGE gel and transferred to Amersham Hybond-ECL nitrocellulose membrane (GE healthcare), according to the manufacturer's instructions. Immunodetection of pADPr was carried out using mouse polyclonal antibody directed against the pADPr (Abcam), followed by anti-mouse horseradish peroxidase-conjugated antibody (Kirkegaard & Perry Laboratories, Inc.). The signal was detected with the Western Lightning Plus-ECL (PerkinElmer).

### Growth inhibition assays

*T. cruzi* epimastigotes were grown in LIT complete medium for 4 days up to a parasite density of 10^7^ parasites/ml. The culture was placed in 96-well sterile plates in 100 µl aliquots and PARP inhibitors were added. Nifurtimox and β-lapachone were used as control compounds. DMSO control was used at 1% v/v concentration. Optical density of the cultures was determined at 600 nm each day for four days to follow parasite growth. Controls for LIT growth medium bearing no parasites were also performed. In all experiments performed, conditions were tested in triplicates. Significance of the results were analysed with one-way ANOVA using GraphPad Prism version 5.03 for Windows (GraphPad Software).

### *Trypanosoma cruzi* infection of Vero and A549 cells

Vero cells were cultured in D-MEM medium (Gibco), supplemented with 2 mM L-glutamine, 10% (v/v) FCS, 100 U/ml penicillin and 100 mg/l streptomycin. The wild type (Sigma-Aldrich) and PARP silenced (sh_PARP) pulmonary adenocarcinoma A549 cell lines were provided by Dr. Virág and Dr. Erdélyi, from the University of Debrecen, Hungary [20]. A549 cells were grown in RPMI-1640 medium (Gibco), supplemented with 2 mM L-glutamine, 1 mM Sodium Pyruvate, 10% (v/v) FCS, 100 U/ml penicillin and 100 mg/l streptomycin.

Trypomastigotes were collected by centrifugation of the supernatant of previously infected cultures at 1500×*g* at room temperature for 7 minutes and incubated for 3 hours at 37°C in order to allow the trypomastigotes to move from the pellet into the supernatant. After this period, the supernatant was collected and trypomastigotes were counted in a Neubauer chamber. The purified trypomastigotes were pre-incubated in the presence or absence of 25 nM Olaparib for 30 minutes and then used to infect new monolayers of Vero or A549 cells. For this, 50 trypomastigotes/cell were added to the medium of 24 hour-old monolayers and incubated for 24 hours at 37°C, after which they were removed by changing the cell culture medium. Infections were performed in the media above indicated, but supplemented with 3% (v/v) FCS.

The infection was allowed to proceed and growth medium was changed periodically during the first 5 days. In the PARP inhibited samples, Olaparib was kept in the growth medium at 25 nM throughout the experiment. At the indicated days, cells were fixed and stained by May Grünwald Giemsa technique. Cells were visualized using an Olympus BX41 microscope. Amastigotes and cells were counted using the ImageJ software in at least 7 microscopic fields. The number of total cells counted per field was between 49 (average at day 2) and 267 (average at day 6). The number of amastigotes counted per field was between 50 (average at day 2) and 869 (average at day 6). Alternatively, trypomastigotes in the supernatant of the cell cultures were counted without prior fixation on a Neubauer Chamber at the indicated days. All experiments were performed in triplicates. Significance of the results were analysed with two-way ANOVA using GraphPad Prism version 5.03 for Windows (GraphPad Software).

## Results

### Activity assay optimization and *in vitro* inhibition of *Trypanosoma cruzi* PARP

The activity assay was optimized for TcPARP by determining the effect of pH, DNA concentration, and various additives to the enzymatic activity. The activity of TcPARP has previously been detected to increase in the presence of DNA strand breaks [7], [8]. The highest activity was achieved by using 20 µg/ml of the DNA in assay buffer. The optimal buffer system was sodium phosphate at pH 7.

A collection of 24 compounds known to improve activities of various enzymes was also assayed on TcPARP activity. Transition metals (MnCl_2_, ZnCl_2_, and NiCl_2_) and detergents (Triton X-100 and IGEPAL) inhibited protein activity, while alkaline earth metals (CaCl_2_ and MgCl_2_), salts (NaCl, KCl, and (NH_4_)_2_SO_4_) and reducing agents (DTT, TCEP) did not have a marked effect on protein activity. Glycerol was identified as the best additive. The final assay buffer consisted of 0.1 M Na_2_HPO_4_/NaH_2_PO_4_ pH 7, 20 µg/ml activated DNA, and 15% glycerol. The assay was also found to tolerate DMSO up to 2% without any decrease in the protein activity. This is well above the concentration of DMSO used in the assay.

**Figure 1:**
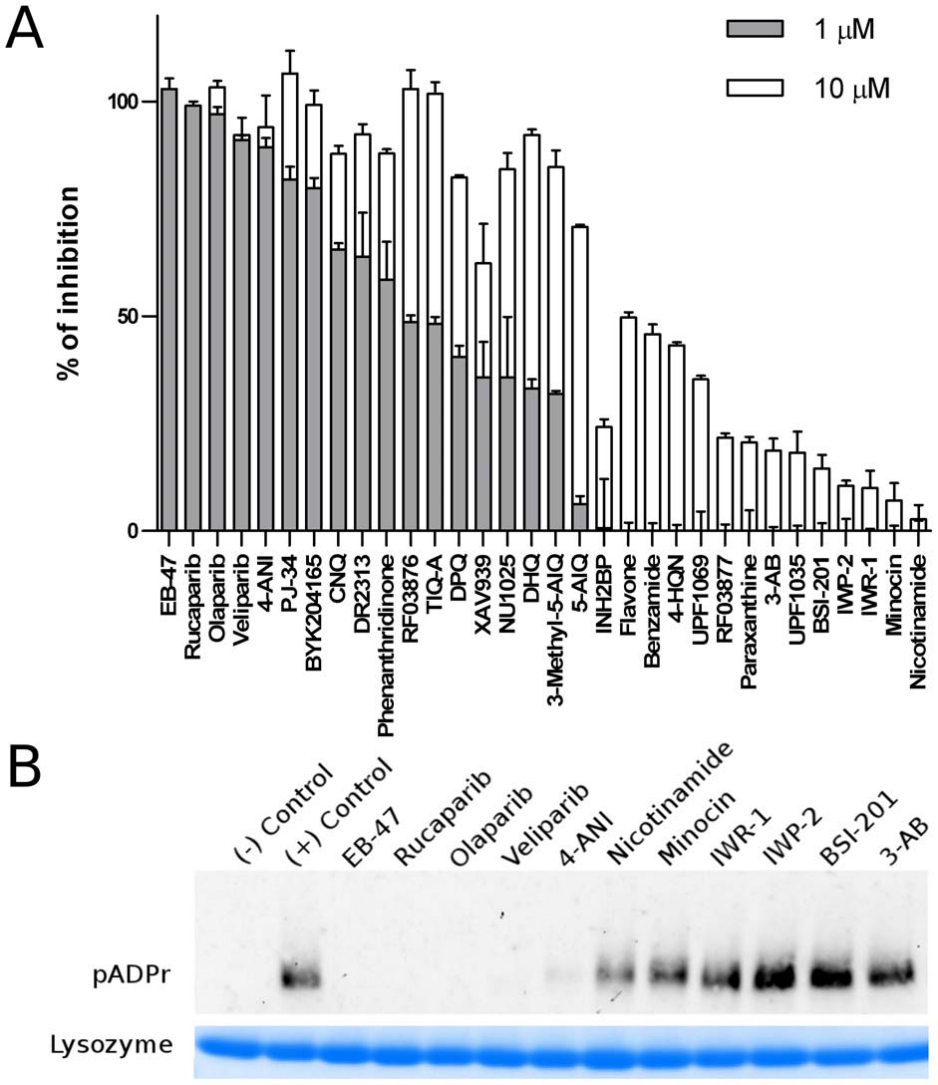
Screening of TcPARP inhibitors. A) A panel of 32 compounds was evaluated for the ability to inhibit TcPARP activity *in vitro*. Compounds were tested at 10 µM and 1 µM concentrations, while protein concentration was 10 nM. The values obtained were converted to % of inhibition in relation to the control performed in the same plate. All data points were determined in duplicates and shown as mean values ±SD. B) Inhibition of TcPARP was confirmed by Western blot using a mixture of biotinylated NAD^+^ (1 µM) and regular NAD^+^ (4 µM) as a substrate. Lysozyme was used as a loading control. TcPARP (150 nM) was incubated in the assay buffer with a set of inhibitors (1 µM). Biotinylated protein was detected by using streptavidin-HRP.

A small in-house library (32 compounds) of known PARP inhibitors was tested for TcPARP inhibition (Table S1). All compounds were analyzed at 10 and 1 µM concentrations (Fig. 1A). Several potent inhibitors were found from the screen and the IC_50_ values of the best inhibitors were determined (Table 1) by the optimized homogeneous activity assay. The most potent inhibitors were Olaparib (IC_50_ = 3.4 nM) [21] and EB-47 (IC_50_ = 8.1 nM) [22], which are also excellent hPARP-1 inhibitors. Notably, many of the compounds were not as potent inhibitors for TcPARP as for hPARP-1, and only EB-47 showed some preference for TcPARP over hPARP-1. The compounds exhibiting the highest inhibition were tested for their ability to impede pADPr formation by Western Blot along with the less potent hits (Fig. 1B). This qualitative assessment confirmed that the compounds selected were indeed able to inhibit the pADPr synthesizing activity of TcPARP.

**Table 1:** IC_50_ values for the best TcPARP inhibitors.

Compound	TcPARP		hPARP-1	Reference
	IC_50_ (µM)	pIC_50_±SE	IC_50_ (K_i_) (µM)	
3-AB	26	4.58±0.094	33	[32]
Phenanthridinone	1.6	5.79±0.089	0.56	[33]
DR2313	1.4	5.86±0.091	0.20	[33]
RF03876	1.3	5.90±0.036	-	-
PJ-34	0.23	6.65±0.107	0.019	[34]
BYK204165	0.18	6.74±0,090	0.045	[34]
CNQ	0.17	6.76±0.075	0.033	[35]
Veliparib	0.083	7.08±0.108	0.005 (K_i_)	[36]
4-ANI	0.043	7.37±0.084	0.022	[34]
Rucaparib	0.025	7.59±0.096	0.0014 (K_i_)	[37]
EB-47	0.0081	8.09±0.069	0.045	[38]
Olaparib	0.0034	8.47±0.071	0.005	[21]

The IC_50_ value of the first generation PARP inhibitor, 3-AB, is measured for reference.

### Structural basis for inhibitor binding and selectivity

**Figure 2:**
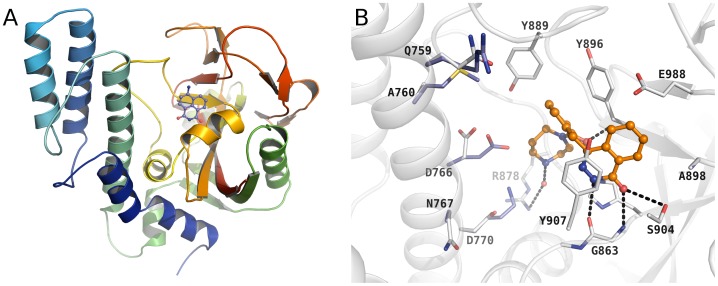
Homology model and inhibitor binding modes. A) Homology model of TcPARP. Also shown is the binding of 4-ANI to the nicotinamide binding site (based on superposition of chicken PARP PDB code 2PAX [31]). B) Active site of hPARP-1 and TcPARP. Conserved residues are shown only for hPARP-1, whereas residues differing in TcPARP are shown in blue. Inhibitor analogous to Olaparib, KU0058948, is shown as it is bound to PARP-3 (PDB code 3C49 [13]).

In order to evaluate the inhibitor selectivity between TcPARP and hPARP-1, we generated an homology model of TcPARP based on the solved human PARP structures. Three of the structures, hPARP-1-3, contain the PARP regulatory (PRD) and the ADP-ribosyl transferase (ART) domains and therefore those were selected for the sequence alignment (Fig. S1). Most of the PARP inhibitors bind to the nicotinamide binding site and the homology model revealed that this region is highly conserved also in TcPARP (Fig. 2A). The most common interactions of the inhibitors (superscript indicates hPARP-1 numbering), such as stacking with the tyrosine^907^, and hydrogen bonds to the glycine^863^ and serine^904^ at the bottom of the nicotinamide cavity, are conserved (Fig. 2B).

The best inhibitors for TcPARP *in vitro* were Olaparib and EB-47. There is no structural information of EB-47 in complex with PARPs, but the structure of Olaparib in complex with human Tankyrase 2 (PARP5b/ARTD6) has recently been solved [23]. However, there are key differences in the nicotinamide binding site between Tankyrase 2 and hPARP-1, specifically in the D-loop (loop lining the donor NAD^+^ binding site), and therefore we used a complex between KU0058948, a close analog of Olaparib, with hPARP-3 (PDB id. 3C49) [13]. Although there are specific differences between hPARP-1 and hPARP-3, the binding mode of the compound is likely very similar. The interactions of the compound with the nicotinamide pocket, the D-loop and Arg400^878^ are conserved and explain the high potency of the compound (Fig. 2B).

EB-47 displayed a higher IC_50_ value than Olaparib, but had approximately five-fold selectivity over hPARP-1. EB-47 is rather large and designed to extend towards the adenosine binding site. There are many residues differing in this region, especially on the side of the regulatory domain (Fig. 2B) and these are likely responsible for the tighter binding of EB-47 to TcPARP. These differences also explain the overall weaker potencies towards TcPARP (Table 1).

### *In vivo* inhibition of *Trypanosoma cruzi* PARP

**Figure 3:**
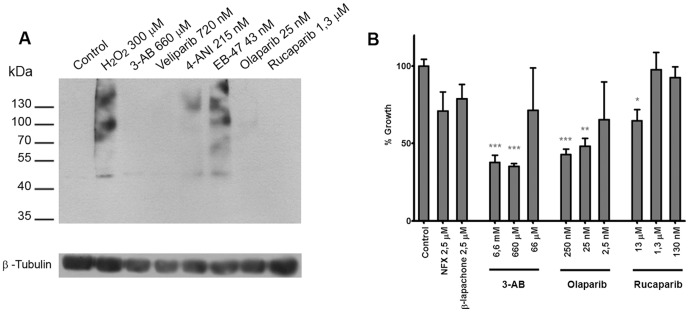
Inhibition of TcPARP *in vivo* and effect of PARP inhibitors on *Trypanosoma cruzi* growth. A) Epimastigotes of *T. cruzi* were pre-incubated with the indicated concentrations of the selected inhibitors for 30 minutes and then subjected to 300 µM hydrogen peroxide for 10 minutes. Amount of pADPr formation was determined on total cell extracts (35 µg) by Western Blot using a mouse monoclonal antibody directed against pADPr. Positive control (H_2_O_2_ 300 µM) was performed in the absence of inhibitor and negative control (Control) was performed on parasites that were not subjected to hydrogen peroxide. A commercial antibody directed against the housekeeping protein β-tubulin was used as a loading control (lower panel). B) Effect of the PARP inhibitors 3-AB, Olaparib and Rucaparib on *T. cruzi* growth was determined by incubating epimastigotes at an initial density of 10^7^ parasites/ml in the continuous presence of inhibitors at the indicated concentrations. Growth in control cultures without TcPARP inhibitors or trypanocidal drugs were set to 100%. All data points were determined in triplicates and shown as means with standard deviation. The result was analyzed with one way ANOVA and the significance versus the control is indicated in the figure (***, p<0.001; **, p<0.01; *, p<0.05).

We reported previously that the low potency PARP inhibitor, 3-AB, was able to reduce *T. cruzi* epimastigote growth when parasites where incubated in the presence of this compound at millimolar concentrations [7]. To further evaluate the compounds we tested their ability to inhibit pADPr formation *in vivo* in *T. cruzi* parasites challenged with 300 µM hydrogen peroxide. The inhibitor concentrations tested *in vivo* were at least five times higher than measured IC_50_ value: Veliparib, 720 nM; 4-ANI, 215 nM; EB-47, 43 nM; Olaparib, 25 nM; and Rucaparib, 1.3 µM. 3-AB was also used as a control at 660 µM concentration. Most of the inhibitors effectively inhibited pADPr formation in the parasites at these concentrations (Fig. 3A). 4-ANI did not show complete inhibition and EB-47 apparently did not inhibit TcPARP at all.

### Effect PARP inhibitors on *Trypanosoma cruzi* epimastigotes

The parasites were grown in the presence of the inhibitors at the same concentrations used for the *in vivo* inhibition assays described above. All of the compounds were able to impact negatively on epimastigote growth in culture, but some of them did it only marginally (Figure S2). DMSO did not affect the growth at 1% v/v (not shown). Therefore, three compounds were selected to be evaluated in a series of concentrations. At the highest concentration tested, all compounds showed a significant inhibition of parasite growth (Fig. 3B). As controls, Nifurtimox (NFX) and β-lapachone, two compounds that have widely been reported to inhibit parasite growth, were also analyzed and they both demonstrated to slow down growth of epimastigotes. The most effective PARP inhibitor tested, Olaparib, diminished epimastigote growth significantly at 25 nM, while β-lapachone, an agent known to cause oxidative damage, and Nifurtimox, the currently used drug in American Trypanosomosis treatment, showed a small effect at 2.5 µM.

### Effect of PARP inhibitors on in vitro infection models

**Figure 4:**
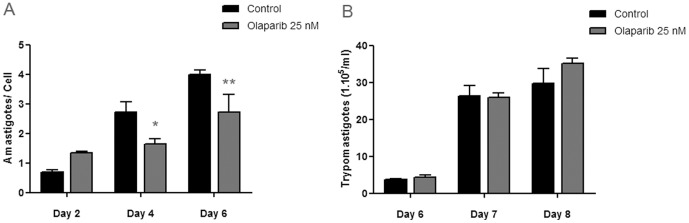
The effect of PARP inhibitors on *Trypanosoma cruzi* infection on Vero cells. *T. cruzi* trypomastigotes were purified from the supernatant of previously infected cultures and preincubated for 30 minutes in D-MEM complete medium in the absence (control) or presence of PARP inhibitor (Olaparib 25 nM). Twenty-four hours Vero cell monolayers were infected with 50 trypomastigotes/cell. The infection process was followed by microscopic direct visualization A) At the indicated days, infected Vero cell monolayers were fixed and a May-Grünwald Giemsa staining was performed in order to visualize intracellular amastigotes. The amastigote/cell ratio was determined by counting the number of amastigotes and cells in several fields. B) The number of trypomastigotes/ml in the supernatant of the infected cell cultures was determined by counting unfixed trypomastigotes in a Neubauer chamber at the indicated days. All data points were determined in triplicates and shown as means with standard deviation. Significance of the result versus the control is indicated (**, p<0.01; *, p<0.05; one way ANOVA).

Because of its high potency, Olaparib was chosen for the analysis of the effect of PARP inhibition on the infection process. In infections carried out using Vero cells, the validated cell line for *Trypanosoma cruzi* infections *in vitro*, the presence of Olaparib (25 nM) in the growth medium lead to a decrease in the number of amastigotes per cell (Fig. 4A). The percentage of infected cells at days 4 and 6 was also lower in Olaparib treated cells when compared to control infections (Table 2). However, there was no difference in the percentage of amastigotes-containing cells at day 2 of the infection, indicating that the initial or primary infection would not be affected by the presence of the PARP inhibitor.

**Table 2:** Percentage of infected Vero cells.

	% of Infected Cells		
	Day 2	Day 4	Day 6
Control	37.20±3.13	20.81±0.64	27.36±2.88
Olaparib 25 nM	38.67±7.68	13.54±3.40*	22.03±2.36*

The percentage of infected cells was determined along the infection process by microscopic visualization and counting after May-Grünwald Giemsa dying. The numbers indicated are the average values with the SD values. Significance of the result versus control is indicated. (*, p<0.05, two way ANOVA).

Interestingly, there was no significant difference in the number of trypomastigotes in the supernatant of these cultures (Fig. 4B). Controls, in which uninfected Vero cells were incubated in the presence or absence of Olaparib, showed that the compound did not alter the growth of the cell line, at least *in vitro* in the conditions tested here.

**Figure 5:**
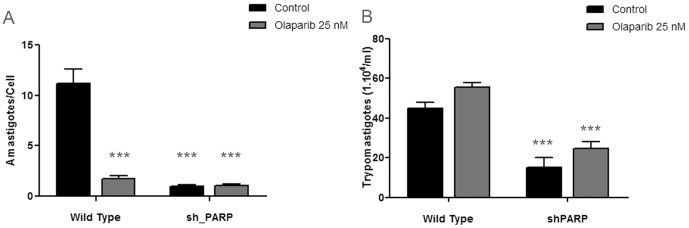
The effect of the absence of PARP activity in the A549 host cell on *Trypanosoma cruzi* infection. *T. cruzi* trypomastigotes were purified from the supernatant of previously infected cultures and preincubated for 30 minutes in RPMI-1640 complete medium in the absence (control) or presence of PARP inhibitor (Olaparib 25 nM). Twenty-four hours A549 wild type or sh_PARP (hPARP-1 silenced) cell monolayers were infected with 50 trypomastigotes/cell. The infection process was followed by microscopic direct visualization A) At day 6 post-infection, infected cell monolayers were fixed and a May-Grünwald Giemsa staining was performed, in order to visualize intracellular amastigotes. The amastigote/cell ratio was determined by counting the number of amastigotes and cells in several fields. B) The number of trypomastigotes/ml in the supernatant of the infected cell cultures was determined at day 9 post-infection by counting unfixed trypomastigotes in a Neubauer chamber. All data points were determined in triplicates and shown as means with standard deviation. Significance of the result versus the Wild type control is indicated (***, p<0.001; two way ANOVA).

Olaparib is also a highly potent hPARP-1 inhibitor. In order to determine whether the reduction in the intracellular amastigotes was due to inhibition of TcPARP or hPARP-1, infection experiments were carried out on monolayers of human A549 cancer cells and on A549 cells where hPARP-1 was silenced by iRNA (sh_PARP). The number of amastigotes per cell in these infections was markedly decreased in wild type cells in the presence of Olaparib. Similar reduction of infection levels was observed in A549 cells where hPARP-1 was knocked down, even in the absence of the PARP inhibiting compound (Fig. 5A). As previously observed for Vero cells infected with *T. cruzi* parasites, there was no significant difference in the number of trypomastigotes in the growth medium of wild type cells in the presence of Olaparib. Interestingly, there was a clear decrease in the trypomastigote number in sh_PARP cell line when compared to the wild type A549 cells (Fig. 5B).

## Discussion

*T. cruzi* contains only one PARP enzyme, which is activated by DNA strand breaks and catalyzes the formation of pADPr [7]. Activation of the human homolog, hPARP-1, leads to the modification of the protein itself and several other proteins involved in the relaxation of the chromatin superstructure, and the recruitment of DNA-break repair enzymes [4]. The PARP-dependent DNA repair processes are not well-understood in *T. cruzi* or other trypanosomatids. Here we present chemical tools for the characterization of ADP-ribose metabolism in *T. cruzi*.

Fluorescence-based activity assay on a 96-well plate showed to be advantageous when compared to the radioactivity-based method previously employed to measure trypanosomatid PARP activity *in vitro*. Besides from the evident safety and cost benefits in the avoidance of the implementation of radioactive reagents, the fluorescence assay developed earlier for hPARP-1 by Putt *et al.* [9] demonstrated to be more reproducible and reduced considerably the amount of sample needed for each individual determination. Due to chemical conversion of NAD^+^, the assay requires basic safety measures to be taken into account. The plate format also allowed higher number of conditions to be tested more efficiently.

Using the optimized activity assay conditions, we identified the best inhibitors for TcPARP from a panel of compounds consisting mostly of known PARP inhibitors. We also verified which of them are able to affect the enzyme activity upon DNA damage *in vivo*. Olaparib, a highly potent hPARP-1 inhibitor, showed to be the most potent inhibitor with an IC_50_ in the low nanomolar range. EB-47 has been reported to inhibit pADPr formation in human cells [19], but apparently this inhibitor was not effectively diminishing pADPr formation in parasites. Partial or complete inability of EB-47 to impede pADPr formation *in vivo* might be related to the impossibility of penetrating *T. cruzi* membrane. A possible explanation for this observation lies in the physical properties of the compound. Membrane penetration and steric factors have been shown to be the key components in drug incorporation to *T. brucei*, a closely related parasite to *T. cruzi* [24]. EB-47 is larger than other active compounds (MW 538 Da) and much more polar than any of the other ones tested with a LogP value of −2.55 (Calculator Plugins, Marvin 5.8.2, 2012, ChemAxon (http://www.chemaxon.com)). This differs from the optimal logP value of 5.8 of drugs targeted towards *T. brucei* [24]. Despite this, EB-47 could be potentially used in the future to achieve greater selectivity for the inhibitors.

Most inhibitors tested here had an impact on *T. cruzi* epimastigotes in culture. 3-AB reduced parasite growth by approximately 60% when compared to the control at concentrations of 660 µM and 6.6 mM, which is in accordance with our previous results [8]. The highest impact on the epimastigotes growth was obtained with one of the newest and most potent PARP inhibitors, Olaparib. When tested at a concentration as low as 25 nM, it led to 50% decrease in growth of the parasites (Fig. 3B). As with 3-AB, raising Olaparib concentration further did not cause a higher impact on parasite growth. Olaparib alone is able to reduce epimastigote growth in culture more than what was observed here for 100 times higher concentration of Nifurtimox (2.5 µM), the drug that has been used for more than 40 years to treat Chagas disease and forms part of a recently approved combination therapy that targets West African trypanosomiasis caused by *T. brucei* [25]. β-lapachone, a drug that together with its derivatives is being proposed as one of the most promising new therapeutical approaches to enter the American Trypanosomiasis treatment scenario [26], has a similar effect as Nifurtimox.

Key steps during infection cycle of *T. cruzi* is the differentiation from the trypomastigote flagellate form to the amastigote intracellular form, as well as the reverse process, from amastigote to trypomastigote. Trypomastigote form allows further re-infections in the patient and causes the persistence and deepening of the disease. Williams has previously reported that millimolar concentrations of first generation PARP inhibitors 3-methoxybenzamide and, to a lesser extent, 3-AB, are able to inhibit differentiation of amastigotes to trypomastigotes [27]. This result was mainly observed for axenic amastigotes cultures (extracellular) and in the presence of high concentrations of the inhibiting compounds. Our results could not confirm conclusively these observations.

Here, the presence of the PARP inhibitor Olaparib reduced the number of intracellular amastigotes in both Vero and A549 cells. The percentage of infected Vero cells was virtually identical between the control experiment and the Olaparib treated cells at the first days of the infection. However, the presence of Olaparib in the culture media lead to a decrease in the fraction of infected cells as the *in vitro* infection progressed temporarily, when compared to the control. The number of trypomastigotes in the growth media was not affected in either case. Experiments carried out in A549 cells where hPARP-1 is absent showed that the host cell PARP has an important role during *T. cruzi* infection. Not only the number of amastigotes per cell was greatly diminished in the hPARP-1 silenced cell line, but there was also a significant reduction in the number of trypomastigotes in the growth medium. These results indicate that, although the importance of TcPARP to the infection process cannot be ruled out, the host PARP activity seems to be participating in the infection process. Recently, Ba and coworkers pointed out that the invasion of cardiac cells in culture by *T. cruzi* trypomastigotes leads to the generation of ROS in the host cell, which are able to cause DNA damage and cause the activation of hPARP-1. This induces the expression of the transcription factor NF-κB [28], which activates the transcription of several cytokines, such as TNF-α and IL-1β. These cytokines collaborate to the invasion process in cultures of human and monkey kidney epithelial cells. Their absence leads to lower levels of *T. cruzi* infections [29]. Additionally, it has been demonstrated that NF-κB acts as a positive regulator of the expression of the cell adhesion molecules ICAM-1, VCAM-1 and selectin-E, which also play a role during the invasion process [30]. It could be that other human PARP isoenzymes play also a role in the process. The observation, that the hPARP-1 knock down had a deeper effect than the treatment with Olaparib however implies that the effect is due to inhibition of hPARP-1 activity and not affected significantly by other human PARP isoenzymes.

The results here observed indicate that the presence of the PARP inhibitor would be affecting at least one of the processes required for a successful infection cycle. The initial infection appears to be unaffected by the presence of the PARP inhibitor here used, since no differences in the percentage of infected cells could be observed during the first days. Even though a possible delay in the duplication time of intracellular amastigotes caused by the presence of Olaparib or an effect of this compound on the amastigotes-to-trypomastigote differentiation cannot be discarded, the results here obtained, taken together with data published by other authors, suggest a possible interference of Olaparib with the generation of the most adequate conditions needed for the persistence of the infection, in which PARP-1 from the host cell would be intimately involved.

The different number of trypomastigotes in PARP inhibited and hPARP-1 silenced cell culture indicates that silencing of hPARP-1 is more effective than inhibition by Olaparib. Because the number of trypomastigotes decreased when hPARP-1 was knocked down but not when PARP activity was inhibited with Olaparib, an activity independent, but hPARP-1 dependent mechanism might be involved in the process. These findings validate further research on the possible use of PARP inhibitors to treat *T. cruzi* infection and the role of parasite and human PARPs in the process. Use of PARP inhibitors such as Olaparib, in human cells, does not have an immediate effect to the healthy cells. In long term they could be harmful to the cells due to the impaired DNA repair mechanism. This should be taken into account and could limit the usefulness of the inhibitors to treat parasite infection.

## Supporting Information

Figure S1 **Sequence alignment of TcPARP together with human hPARP-1-3.** Alignment covers C-terminal PRD and ART domains. PDB codes used to adjust the alignment are indicated for hPARP-1-3. Sequence numbers and the secondary structure above correspond to hPARP-1. Conserved HYE motif is marked with circles and the residues lining the NAD^+^ binding site that differ between PARP-1 and TcPARP are marked with triangles.
(TIF)

Figure S2 **Effect of PARP inhibitors not shown in** **Fig. 4** **on** ***Trypanosoma cruzi*** **epimastigote growth.** Effect of Veliparib, 4-ANI and EB-47 on *T. cruzi* growth was determined by incubating epimastigotes at an initial density of 10^7^ parasites/mL in the continuous presence of inhibitors at the following concentrations: Veliparib 720 nM, 4-ANI 215 nM and EB-47 43 nM. All data points were determined in triplicates and shown as means with standard deviations.
(TIF)

Table S1 Details of the PARP inhibitor like compounds tested.
(DOCX)
