# Supplementary figures and images for: Inhibition of poly(ADP-ribose) Polymerase Interferes with Trypanosoma cruzi Infection and Proliferation of the Parasite
Source: PLoS One. 2012 Sep 25;7(9):e46063. doi: 10.1371/journal.pone.0046063 (PMC3457943; doi:10.1371/journal.pone.0046063)

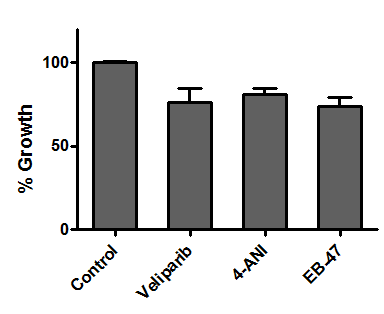

Supplement: Figure S2 — Effect of PARP inhibitors not shown in Fig. 4 on Trypanosoma cruzi epimastigote growth. Effect of Veliparib, 4-ANI and EB-47 on T. cruzi growth was determined by incubating epimastigotes at an initial density of 107 parasites/mL in the continuous presence of inhibitors at the following concentrations: Veliparib 720 nM, 4-ANI 215 nM and EB-47 43 nM. All data points were determined in triplicates and shown as means with standard deviations. (TIF) [file pone.0046063.s002.tif]
