# Supplementary material for: Inhibition of poly(ADP-ribose) Polymerase Interferes with Trypanosoma cruzi Infection and Proliferation of the Parasite
Source: PLoS One. 2012 Sep 25;7(9):e46063. doi: 10.1371/journal.pone.0046063 (PMC3457943; doi:10.1371/journal.pone.0046063)
Supplement: Table S1 — Details of the PARP inhibitor like compounds tested. (DOCX) [file pone.0046063.s003.docx]

**Supplementary Table 1.** PARP inhibitor like compounds tested.

| **No.** | **Compound name (IUPAC)** | **Acronyms** | **Supplier** |
| --- | --- | --- | --- |
| 1 | 3-aminobenzamide | 3AB | Alexis Biochemicals |
| 2 | Nicotinamide | Niacinamide | Alexis Biochemicals |
| 3 | Benzamide |  | Alexis Biochemicals |
| 4 | 2-[(2R)-2-methylpyrrolidin-2-yl]-1H-1,3-benzodiazole-4-carboxamide | Veliparib | Alexis Biochemicals |
|  |  | ABT-888 |  |
| 5 | 5-amino-1,2-dihydroisoquinolin-1-one | 5-aminoisoquinolinone | Alexis Biochemicals |
|  |  | 5-AIQ |  |
| 6 | 5-amino-3-methyl-1,2-dihydroisoquinolin-1-one | 3-methyl-5-aminoisoquinolinone | Alexis Biochemicals |
|  |  | 3-methyl-5-AIQ |  |
| 7 | 8-amino-3-azatricyclo[7.3.1.0^{5,13}]trideca-1(12),5,7,9(13),10-pentaene-2,4-dione | 4-amino-1,8-naphthalimide | Alexis Biochemicals |
|  |  | 4-ANI |  |
| 8 | 5-[4-(piperidin-1-yl)butoxy]-1,2,3,4-tetrahydroisoquinolin-1-one | DPQ | Alexis Biochemicals |
| 9 | 2-methyl-1H,4H,5H,7H,8H-thiopyrano[4,3-d]pyrimidin-4-one | DR2313 | Alexis Biochemicals |
| 10 | 2-(4-{[(2S,3S,4R,5R)-5-(6-amino-9H-purin-9-yl)-3,4-dihydroxyoxolan-2-yl]carbonyl}piperazin-1-yl)-N-(1-oxo-2,3-dihydro-1H-isoindol-4-yl)acetamide | EB-47 | Alexis Biochemicals |
| 11 | 1,4-dihydroquinazolin-4-one | 4-Hydroxyquinazoline | Alexis Biochemicals |
|  |  | 4-HQN |  |
| 12 | 6-amino-5-iodo-2H-chromen-2-one | INH2BP | Alexis Biochemicals |
| 13 | 5-hydroxy-1,2-dihydroisoquinolin-1-one | 1,5-Isoquinolinediol | Alexis Biochemicals |
|  |  | DHQ |  |
| 14 | (2E,4S,4aS,5aR,12aS)-2-[amino(hydroxy)methylidene]-4,7-bis(dimethylamino)-10,11,12a-trihydroxy-1,2,3,4,4a,5,5a,6,12,12a-decahydrotetracene-1,3,12-trione | Minocin | Alexis Biochemicals |
|  |  | Minocycline |  |
| 15 | 8-hydroxy-2-methyl-1,4-dihydroquinazolin-4-one | NU1025 | Alexis Biochemicals |
| 16 | 5,6-dihydrophenanthridin-6-one | Phenanthridinone | Alexis Biochemicals |
| 17 | 2-(dimethylamino)-N-(6-oxo-5,6-dihydrophenanthridin-2-yl)acetamide | PJ-34 | Alexis Biochemicals |
| 18 | 4H,5H-thieno[2,3-c]isoquinolin-5-one | TIQ-A | Alexis Biochemicals |
| 19 | 1,7-dimethyl-2,3,6,7-tetrahydro-1H-purine-2,6-dione | Paraxanthine | Sigma-Aldrich |
|  |  | 1,7-dimethylxanthine |  |
| 20 | 3-(4-chlorophenyl)quinoxaline-5-carboxamide | CNQ | Calbiochem |
| 21 | 4-({3-[(4-cyclopropanecarbonylpiperazin-1-yl)carbonyl]-4-fluorophenyl}methyl)-1,2-dihydrophthalazin-1-one | Olaparib | JS Research Chemicals Trading |
|  |  | KU-0059436 |  |
|  |  | AZD-2281 |  |
| 22 | (4Z)-4-[(1-methyl-1H-pyrrol-2-yl)methylidene]-1,2,3,4-tetrahydroisoquinoline-1,3-dione | BYK204165 | Sigma-Aldrich |
| 23 | 2-[4-(trifluoromethyl)phenyl]-1H,4H,5H,7H,8H-thiopyrano[4,3-d]pyrimidin-4-one | XAV939 | Maybridge |
| 24 | 2-(pyridin-2-yl)-5H,7H,8H-thiopyrano[4,3-d]pyrimidin-4-ol | RF03877 | Maybridge |
| 25 | 2-cyclopropyl-5H,7H,8H-thiopyrano[4,3-d]pyrimidin-4-ol | RF03876 | Maybridge |
| 26 | 4-[(1R,2S,6R,7S)-3,5-dioxo-4-azatricyclo[5.2.1.0^{2,6}]dec-8-en-4-yl]-N-(quinolin-8-yl)benzamide | IWR-1 | Sigma-Aldrich |
| 27 | N-(6-methyl-1,3-benzothiazol-2-yl)-2-({4-oxo-3-phenyl-3H,4H,6H,7H-thieno[3,2-d]pyrimidin-2-yl}sulfanyl)acetamide | IWP-2 | Sigma-Aldrich |
| 28 | 4-iodo-3-nitrobenzamide | Iniparib | Selleck Biochemicals |
|  |  | BSI-201 |  |
| 29 | 6-fluoro-2-{4-[(methylamino)methyl]phenyl}-3,10-diazatricyclo[6.4.1.0^{4,13}]trideca-1,4(13),5,7-tetraen-9-one | AG014699 | Selleck Biochemicals |
| 30 | 1-oxo-1,2-dihydroisoquinolin-5-yl benzoate | UPF1035 | Alexis Biochemicals |
| 31 | 5-(2-oxo-2-phenylethoxy)-1,2-dihydroisoquinolin-1-one | UPF1069 | Alexis Biochemicals |
| 32 | 2-phenyl-4H-chromen-4-one | Flavone | Sigma-Aldrich |
